# Cardiogenic shock: incidence, survival and mechanical circulatory support usage 2007–2017-insights from a national registry

**DOI:** 10.1007/s00392-020-01781-z

**Published:** 2020-11-30

**Authors:** Corinna N. Lang, Klaus Kaier, Viviane Zotzmann, Peter Stachon, Torben Pottgiesser, Constantin von zur Muehlen, Manfred Zehender, Daniel Duerschmied, Bonaventura Schmid, Christoph Bode, Tobias Wengenmayer, Dawid L. Staudacher

**Affiliations:** 1 Heart Center Freiburg University, Department of Cardiology and Angiology I, Faculty of Medicine, University of Freiburg, Hugstetter Str. 55, 79106 Freiburg, Germany; 2 Department of Medicine III (Interdisciplinary Medical Intensive Care), Medical Center, Faculty of Medicine, University of Freiburg, Freiburg, Germany; 3 Faculty of Medicine, Institute for Medical Biometry and Statistics, University of Freiburg, Freiburg, Germany; 4 Department of Emergency Medicine, University Hospital of Freiburg, Faculty of Medicine, University of Freiburg, Freiburg, Germany

**Keywords:** Cardiogenic shock, ECMO, pVAD, Mechanical circulatory support

## Abstract

**Background:**

A central element in the management of cardiogenic shock (CS) comprises mechanical circulatory support (MCS) systems to maintain cardiac output (CO). This study aims to quantify incidence, outcome and influence of MCS in CS over the last decade.

**Methods:**

All patients hospitalized with CS in a tertiary university hospital in Germany between 2007 and 2017 were identified utilizing the international coding system ICD-10 with code R57.0. Application of MCS was identified via German procedure classification codes (OPS).

**Results:**

383,983 cases of cardiogenic shock were reported from 2007 to 2017. Patients had a mean age of 71 years and 38.5% were female. The incidence of CS rose by 65.6% from 26,828 cases in 2007 (33.1 per 100,000 person-years, hospital survival 39.2%) to 44,425 cases in 2017 (53.7 per 100,000 person-years, survival 41.2%). In 2007, 16.0% of patients with CS received MCS (4.6 per 100,000 person-years, survival 46.6%), dropping to 13.9% in 2017 (6.6 per 100,000 person-years, survival 38.6%). Type of MCS changed over the years, with decreasing use of the intra-aortic balloon pump (IABP), an increase in extracorporeal membrane oxygenation (VA-ECMO) and percutaneous ventricular assist device (pVAD) usage. Significant differences regarding in-hospital survival were observed between the devices (survival: overall: 40.2%; medical treatment = 39.5%; IABP = 49.5%; pVAD = 36.2%; VA-ECMO = 30.5%; *p* < 0.001).

**Conclusions:**

The incidence of CS is increasing, but hospital survival remains low. MCS was used in a minority of patients, and the percentage of MCS usage in CS has decreased. The use rates of the competing devices change over time.

**Graphical Abstract:**

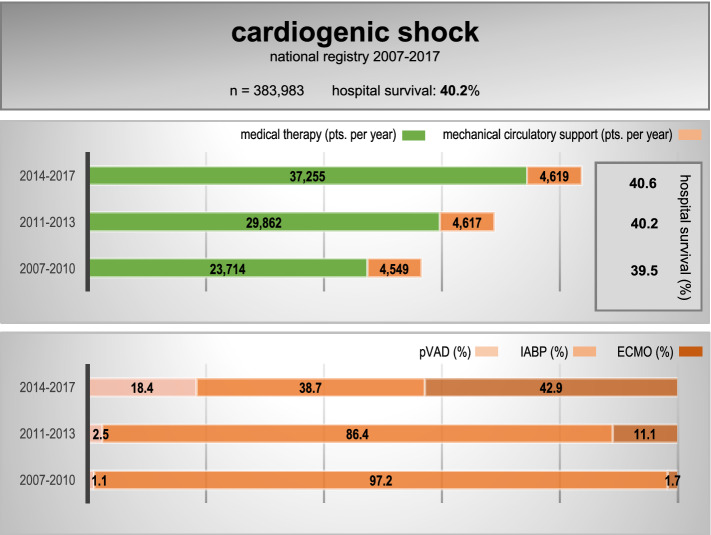

**Electronic supplementary material:**

The online version of this article (10.1007/s00392-020-01781-z) contains supplementary material, which is available to authorized users.

## Introduction

Oxygen supply and oxygen demand are mismatched in cardiogenic shock (CS) as the most severe manifestation of acute heart failure due to an impaired cardiac output (CO). Besides contractile dysfunction of the left, right or both ventricles, a severe end-organ hypoperfusion results in multi-organ failure syndrome occasionally accompanied by a systemic inflammatory response [1].

Acute coronary syndrome (ACS) is the underlying major pathology of CS (up to 80% of the cases) [2]. Furthermore, up to 13% of acute myocardial infarction (AMI) manifests with CS [3]. Rare findings are mechanical complications of AMI, such as ventricular septum defect (VSD), rupture of the free wall or papillary muscle rupture with subsequent severe acute mitral regurgitation. Recent papers hint at present high numbers of non-ischemic shock scenarios (up to 52%)[4]. They may result from pulmonary embolism, pericardial tamponade, myocarditis, arrhythmia, valvular disease, decompensated congestive heart failure or other cardiomyopathies (peripartal, autoimmune, stress induced) [5, 6].

Resolving the underlying cause in acute myocardial infarction complicated by CS is crucial: a revascularization delay of only 10 min results in significantly higher death rates (3.3 additional deaths for every 10 min delay) [7, 8]. The advances in survival (from formerly 30% to nowadays about 60%) [9] can clearly be attributed to rapid percutaneous revascularization, enhanced revascularization techniques (culprit lesion only strategy [10, 11], radial access site [12]), and highly effective platelet therapy—conforming to current European Guidelines [13, 14].

Differential inotropic strategies (dobutamine, dopamine, adrenalin) were adopted to oppose refractory low CO, but resulted in higher mortality rates, presumably due to higher oxygen consumption [15].

The goal of short-term MCS is to assist or to take over CO, and to sufficiently sustain organ perfusion, ideally without further harming the heart. The damaged heart is set at rest until recovery or until a permanent solution is obtained (i.e., LVAD or heart transplantation).

Sophisticated technologies to maintain temporary cardiac salvage mainly consist of percutaneous vascular access and different intra- or extracorporeal pump systems, allowing rapid access and a broad use [16].

IABP was the first broadly used support system. With technical progress, VA-ECMO became usable outside of heart surgery settings in the intensive care units and recently as resuscitation tools for refractory out-of-hospital cardiac arrest.

MCS strategies have evolved over the past two decades to act as bridging instruments in acute heart failure, either as bridges to recovery/decision, bridges to LVAD or heart transplantation, and in rare cases after extracorporeal CPR bridge to organ donation.

Guidelines do not recommend IABP therapy in CS after AMI following two landmark studies discouraging a broader use of IABP in these patients [14, 17, 18].

A small prospective and a larger retrospective study comparing Impella (pVAD) with IABP-guided therapy in cardiogenic shock showed comparable mortality rates (50 vs. 46%; *p* = 0.92 and 48.5 vs. 46.4%, *p* = 0.64). A higher incidence of bleeding and vascular entry site lesions complicated Impella therapy in both studies [19, 20].

VA-ECMO is increasingly adopted in Germany despite the lack of evidence and neutral IABP studies [21].

Mortality data are derived from registries such as the Extracorporeal Life Support Organization (ELSO), always inheriting the risk of selective reporting. Long-term data on quality of life after CS and MCS are scarce but promising [22–24].

In this manuscript, we present the incidence and mortality of CS in Germany over 10 years considering different treatment protocols and competing MCS systems in the face of poor prior evidence.

## Methods

Since 2005, data on all hospitalizations in Germany have been available for scientific use via the diagnosis-related group (DRG) statistics collected by the Research Data Center of the Federal Bureau of Statistics (DESTATIS). The World Health Organization published the 10th revision of the international statistical classification of diseases and related-health problems (ICD) in January 1998. These hospitalization data, including diagnoses and procedures, are a valuable source of representative nationwide data on in-hospital treatment of patients.

This database represents a virtually complete collection of all hospitalizations in German hospitals that are reimbursed according to the DRG system. From this database, we extracted data on all patients that were hospitalized between 2007 and 2017 with documented cardiogenic shock (ICD-10 code R57.0 as main or secondary diagnosis). Utilization of mechanical circulatory support was identified using the German Procedure Classification/OPS code 8-83a3* (pVAD/Impella), 8-83a0* (IABP), and 8-8523 (VA-ECMO).

Our study did not involve direct access by the investigators to data on individual patients, but only access to summary results provided by the Research Data Center. Therefore, approval by an ethics committee and informed consent were determined not to be required in accordance with German law. All summary results were anonymized by DESTATIS. In practice, this means that any information used in reaching conclusions regarding a single patient or a specific hospital is censored by DESTATIS to guarantee data protection. Especially, the use of the anonymous, persistent ‘institute indicator of hospitals’ is highly restricted in order not to publish any information directly attributable to a single hospital.

The primary outcome was in-hospital mortality which is part of DESTATIS own set of variables. A number of patient characteristics (defined by Reinöhl et al. [25]) are shown in the supplemental appendix. Incidence of cardiogenic shock per 100,000 person-years was approximated by dividing the number of patients with CS by the number of inhabitants of Germany in the respective year according to the Federal Statistical Office of Germany´s estimate [26]. Differences in in-hospital mortality between groups were calculated with the Chi-square test. Trends in in-hospital mortality over time were estimated by means of logistic-regression analyses with time as the sole covariate. Analyses were carried out with the use of Stata software, version 16 (StataCorp, College Station, USA) and Prism, version 5 (GraphPad, San Diego, USA).

## Results

### Patient collective and incidence of CS

A total of 383,983 patients with reported CS could be identified within the analyzed time period from 2007 to 2017. Patients had a mean age of 71 years (on MCS 66 years) and 38.5% were female. Patients’ characteristics are given in Table 1. Additional details including subgroups of MCS are given in the electronic supplemental material (ESM, Supplemental Table 1).

**Table 1:** Baseline characteristics and comorbidities of all patients with cardiogenic shock 2007—2017

	Total	Medical therapy	Mechanical circulatory support
*N*	383,983	333,459	50,524
Age in years	71.3	72.2	65.5
Female gender	147,711 (38.5%)	133,426 (40.0%)	14,286 (28.3%)
CAD	179,740 (46.8%)	140,581 (42.2%)	39,159 (77.5%)
Hypertension	141,339 (36.8%)	121,935 (36.6%)	19,404 (38.4%)
Previous AMI	25,562 (6.7%)	22,016 (6.6%)	3546 (7.0%)
Previous CABG	24,102 (6.3%)	20,506 (6.2%)	3596 (7.1%)
Previous cardiac surgery	33,448 (8.7%)	28,661 (8.6%)	4787 (9.5%)
Peripheral vascular disease	28,254 (7.4%)	24,339 (7.3%)	3915 (7.8%)
Carotid disease	6236 (1.6%)	4968 (1.5%)	1268 (2.5%)
COPD	38,772 (10.1%)	35,072 (10.5%)	3700 (7.3%)
Pulmonary hypertension	28,302 (7.4%)	23,563 (7.1%)	4739 (9.4%)
Renal disease GFR < 15%	14,860 (3.9%)	13,611 (4.1%)	1249 (2.5%)
Renal disease GFR < 30%	20,210 (5.3%)	18,575 (5.6%)	1635 (3.2%)
Atrial fibrillation	121,074 (31.5%)	105,179 (31.5%)	15,895 (31.5%)
Diabetes	116,314 (30.3%)	100,872 (30.3%)	15,442 (30.6%)

Presentation of baseline characteristics and comorbidities of all patients with cardiogenic shock 2007–2017 for the whole cohort, and grouped in medically treated or on mechanical circulatory support (MCS). The total case number is followed by the percentage

**Table 2:** Incidence and survival in reported cardiogenic shock 2007–2017

	2007–2010		2011–2013		2014–2017		Total 2007–2017	
	*N*	Survival	*N*	Survival	*N*	Survival	*N*	Survival
Whole cohort	113,053	39.5%	103,437	40.2%	167,493	40.6%	383,983	40.2%
Medical therapy	94,856	37.6%	89,585	39.2%	149,018	40.8%	333,459	39.5%
Any MCS	18,197	49.3%	13,852	46.9%	18,475	38.7%	50,524	44.8%
pVAD	196	33.2%	348	29.6%	3,401	37.0%	3,945	36.1%
IABP	17,693	49.9%	11,969	49.7%	7,143	48.3%	36,805	49.5%
VA-ECMO	308	25.6%	1,535	29.3%	7,931	30.9%	9,774	30.5%

The case numbers of cardiogenic shock incidence from 2007 to 2010, 2011 to 2013 and 2014 to 2017 as well as the total case numbers from 2007 to 2017 are shown. A constant rise of case numbers of cardiogenic shock is depicted. Additionally, the survival of the different cohorts is displayed in percentage grouped in medical or MCS therapy with subgroups of pVAD, IABP and VA-ECMO

**Fig. 1:**
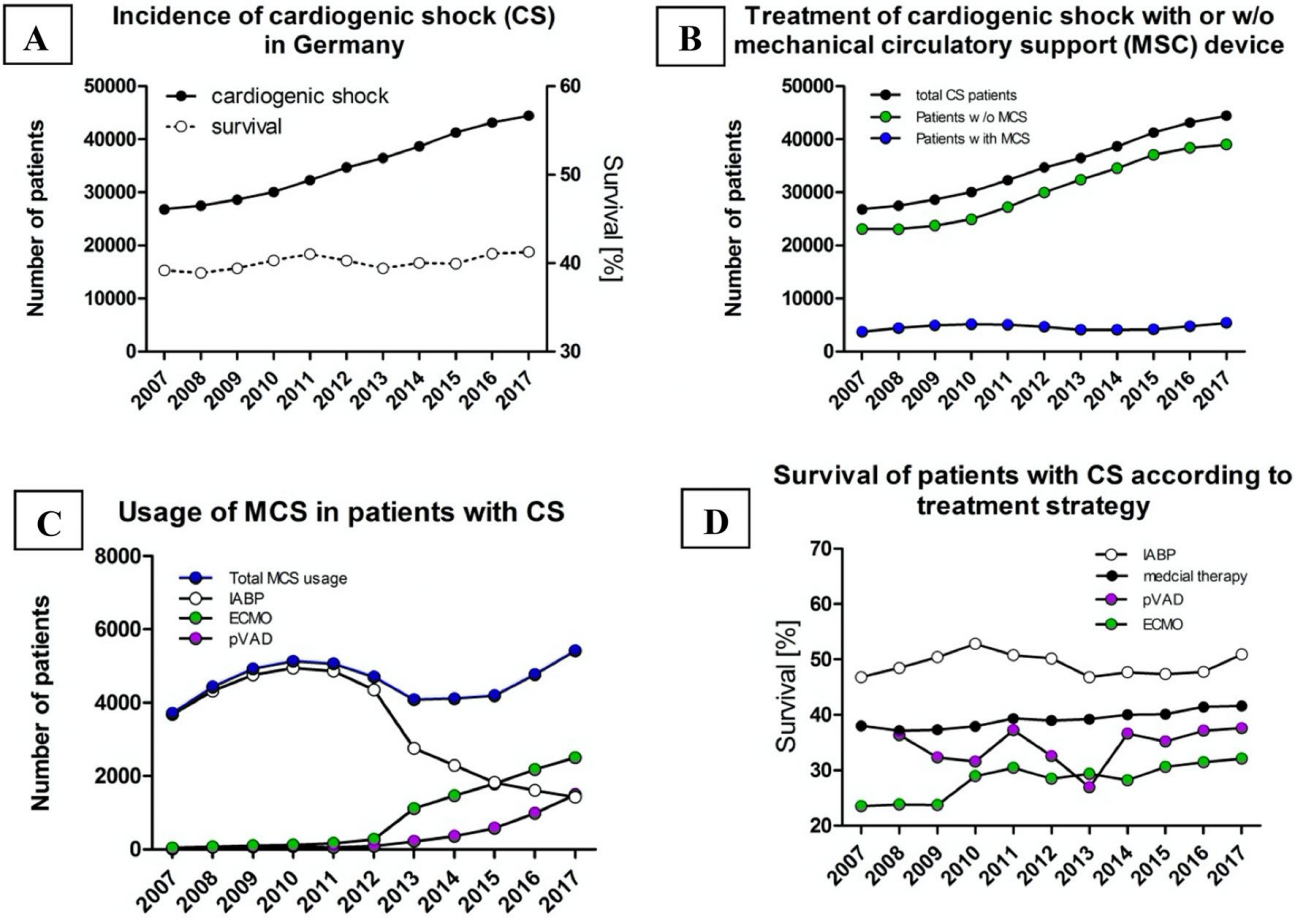
Incidence, survival of cardiogenic shock and mechanical circulatory support usage. **a** An increasing incidence (black) of reported cardiogenic shock in Germany is depicted from 2007 to 2017 with a low overall survival (white) at 40.2% and a very small increase of 2.1% over the last decade in total. **b** The increasing numbers of cases of cardiogenic shock in total (black) in Germany is presented from 2007 to 2017. Whereas the total number of cardiogenic shock and medically treated patients (green) is rising, the percentage of mechanical circulatory support (blue) is declining. **c** The number of patients who were assisted with different mechanical circulatory support (MCS) systems are displayed in Germany from 2007 to 2017, including the total number on MCS (blue), intraaortic balloon pump (IABP; white), percutaneous ventricular assist device (pVAD = Impella; violet) and veno-arterial extracorporeal membrane oxygenation (VA-ECMO; green). The sharp decline in IABP usage in 2013 is followed by a decrease in the total usage. In the year 2013, pVAD and VA-ECMO therapies started rising and provide for an increase in total MCS usage until 2017. Nevertheless, IABP remained more frequent until 2015. Afterwards VA-ECMO therapy takes over as most common device. **d** The usage of different MCS devices result in different hospital survival rates in Germany from 2007 to 2017. Hospital survival of patients on VA-ECMO (green) in cardiogenic shock is lowest, but rising steadily over the last years. It is followed by pVAD (violet) therapies. The application of IABP (white) therapy shows the best survival (even better than medical treatment only strategy; black)

The number of patients with reported CS increased from 26,828 in 2007 (33.1 per 100,000 person-years) to 34,670 in 2012 (43.1 per 100,000 person-years) to 44,425 cases in 2017 (53.7 per 100,000 person-years) in 2017 (Fig. 1a).

### MCS usage

The total number of MSC usage increased as well as the variety of systems grew (from 3710 implantations in 2007 to 5415 implantations in 2017). Nevertheless, the rate of MCS usage in CS decreased by relatively 13.5% (absolute 2.1%) from 16.0% (2007) to 13.9% (2017), when we consider the steep rise of reported CS of relative 65.6% from 26,828 (2007) to 44,425 (2017) (see Fig. 1b).

There was a significant shift in the type of MCS used over the observed time period. While in 2007 virtually all MCS implanted were IABP, their utilization peaked in 2010 and decreased significantly in 2013. This decrease in IABP usage was compensated by an increase in pVAD and VA-ECMO usage (Table 2). In 2017, VA-ECMO was the most commonly used MCS with implantation in 5.6% of all CS patients, followed by pVAD in 3.4% (Fig. 1c, Supplemental Table 2).

### Survival

The overall hospital survival of patients with reported CS between 2007 and 2017 was 40.2%. We could detect a small, but steady increase in hospital survival from 39.2% in 2007 to 41.2% in 2017 (Fig. 1d). Considering the increase in survival of 2.1% (between 2007 and 2017) and the total number of patients with CS of 44,425 in 2017, a total of 915 patients survived in 2017, who would otherwise have died in 2007.

Taking into account only the patients without any MCS, we found the same steady increase in incidence of reported CS from 23,118 in 2007 to 39,010 in 2017. Survival increased even more markedly from 38.0% in 2007 to 41.6% in 2017, an absolute increase of 3.6% or 1,413 additional patient lives saved in 2017 (Supplemental Table 2).

Survival of patients on MCS was significantly different between the devices (survival: IABP = 49.5%; pVAD = 36.2%; VA-ECMO = 30.5%; *p* < 0.001). Highest overall survival was detected in patients with IABP. Survival increased over the observational period for patients with pVAD (*p* = 0.030) and VA-ECMO (p = 0.001) and remained steady for patients with IABP (*p* = 0.586).

## Discussion

We provide epidemiological descriptive data on the incidence, real-life usage of different MCS systems and survival in CS over the last decade in a European high-income country. To note, survival data cannot be derived for evidence for superiority or minority of different MCS systems.

### Incidence of CS

We found a significant increase in the incidence of reported CS. Our findings are in line with similar increases in recent smaller registries from London, UK [27], or Bremen, Germany [28]. However, they diverge from nationwide French or Swiss registries, which find decreasing or stable incidences of CS [9, 29]. However, the latter cover an earlier time period. In Germany, cardiovascular disease is the number one cause of death, with 344,500 (37%) deaths in 2017 (compared to deaths due to cancer at 30%, deaths due to traffic accidents at 0.4%) [26]. In view of our findings that 18,300 deaths are due to CS in 2017, CS causes death in 5.3% of cardiac death cases, and 1.9% of all deaths in Germany.

Due to the nature of this research, we cannot rule out that the increase in reported number of CS is caused by increased coding. There is only one code for CS in the ICD-10 code, which eliminates the confounder of overlap or double hits. Since hospital survival rate is not dramatically improving, a significant over-coding of patients without CS seems implausible.

### MCS usage

The type of MCS used has significantly changed over the last decade. In 2007, the predominant MCS used was IABP. A sharp decline in the utilization of this formerly, most frequently used method is seen in 2013 and may be attributable to the IABP shock trial which was negative for the use of IABP in cardiogenic shock [17]. Other MCS have not been validated in prospective randomized trials. Therefore, it might be reasonable that the percentage of patients with MCS in CS decreased over the last decade. Our data from Europe is in agreement with data from the USA from 2004 to 2014, which reported a steeper increase in CS incidence than in MCS usage [30]. VA-ECMO as well as pVAD compensated for the reduced usage of IABP by 2013. Selection and use of MCS devices may have been biased by local differences in patient selection for MCS, equipment, resources, operator training and economic considerations. Since sicker patients might have been put on VA-ECMO as rescue therapy, survival differences seen for each MCS cannot be used as surrogate for effectiveness in treatment of cardiogenic shock based on our data.

### Survival of CS

Hospital survival in patients with reported CS remains low at 40.2%, with a very small increase of 2.1% over the last decade. Our observation is in line with literature that reports an in-hospital survival rate in CS of around 30% in the 1980s, a rate which has increased and tapered to around 40% in the last years [3].

Tremendous efforts have been undertaken in improving the outcome of CS over the last decade and might be responsible for the improvement. Due to the descriptive nature of the data presented here, we can only speculate on which steps might have been associated with outcomes.

First, early revascularization in CS due to AMI (suggested Class 1B recommendation in current ESC guidelines) is a cornerstone of improved survival [14]. It has been demonstrated elegantly that especially in patients with ST-elevation myocardial infarction and CS, delayed revascularization results in inferior outcomes [7] and that culprit-only percutaneous coronary intervention might be superior to a complete revascularization [11].

High hopes were placed on the implementation of MCS in CS [31]. Our data suggest that MCS usage in everyday care, however, is limited to a minority of patients with CS. Hospital survival rate of patients with (any) MCS is higher than in patients without (survival: any MCS = 44.8%; medical treatment = 39.5%). Interestingly, leaving out IABP, hospital survival with pVAD or VA-ECMO is lower (suggesting a use as salvage therapy). Importantly, we report a significant difference in outcome connected to the different MCS used. VA-ECMO had the lowest survival rate (30.5%) compared to the other MCS devices. The presented VA-ECMO survival data are lower than the overall hospital survival reported by the international ELSO registry (42% survival to discharge or transfer) in adult VA-ECMO in CS. ELSO data have been used for derivation of the SAVE-Score (survival after veno-arterial ECMO) [32]. The international ELSO registry lists 2492 total VA-ECMO runs in CS from 1986 to 2016 with a survival of 41%; the present data identifies four times as many cases (9774 total VA-ECMO runs) from 2007–2017 with a survival of 30%—nevertheless, we cannot avoid a possible bias of previous CPR situations in this analysis.

The Impella-EUROSHOCK-registry reports a 30-day mortality of 64.2% in CS and Impella 2.5 (*N* = 120) [33]. These results are in line with our results with 3945 pVAD runs.

The hospital survival rates of patients with IABP were significantly higher than for patients with other MCS or for patients with medical therapy. A recent retrospective study from the USA reported a higher risk between 2015 and 2017 of in-hospital death and major bleeding among 1,680 pVAD compared to matched pairs of IABP-supported patients in CS following myocardial infarction [34]. These exciting findings, together with older data from IABP trials [35], might trigger more research with IABP to determine if there is a patient collective which might benefit from a counter pulsation.

Improved medical treatment on the intensive care units comprises hemodynamic stabilization by fluid resuscitation, vasopressors and inotropic agents, as well as additional treatments for liver and renal failure.

In chronic heart failure, recent pharmaceutic advantages are implemented into daily routine following expert consensus (sodium–glucose co-transporter 2 inhibitors, sacubitril/valsartan). Sacubitril/valsartan might also play a role in new-onset heart failure without CS [36]. In acute heart failure, pharmaceutics are still in the experimental state, such as omecamtiv mecarbil (GALACTIC-HF), ularitide (TRUE-HF), serelaxin (RELAX-HF-2), tolvaptan (EVEREST-HF), SERCA-2a gene modulation (CUPID-2b) [36, 37]. The use of diuretics is indisputable in patient with signs of fluid overload and vasodilators in acute heart failure with a systolic blood pressure > 90 mmHg. Inotropic agents can be considered for short-term use in case of hypotension or end-organ dysfunction. Additionally, vasopressors might be necessary [38]. Out of a large number of pharmaceutics designed for acute heart failure, levosimendan remains the only potentially useful drug, currently reaching only a low level of evidence [39]. The SURVIVE study reported about a beneficial effect of levosimendan in 2007 [40], followed by meta-analyses from 2012 to 2015 underpinning the positive outcome. In 2017, levosimendan did not prove to be superior to standard care in cardiac surgery [41]. PDE III inhibitors are only considered to reverse beta-blocker effects and did not prove superior to standard care in 2002 [42].

To improve quality, standardize therapeutic strategies and lower mortality in cardiogenic shock, in 2016 the German cardiology society (DGK) encouraged building highly specialized units for heart failure (HFU) analog to chest pain units (CPU) in acute myocardial infarction. Thus, the first CPUs were certified in 2008 for the improved care of patients with acute myocardial infarction or alternative diagnosis (so far, 292 CPUs have been introduced; https://cpu.dgk.org last accessed 08/11/2020). The positive impact of these units has not been proven decisively, as study results are conflicting [43, 44]. Later on, the first HFU was introduced in 2012 in Heidelberg [45, 46]. Until today, 32 supraregional HFU next to 37 regional HFU have been established (https://hfu.dgk.org, last accessed 08/11/2020). A relevant number of patients with heart failure (up to 25% within 1 year) die after hospitalization [47]. This is why the DGK promotes HF-networks (HF-NET) with physicians and nurses in the ambulatory sector. The impact of HFU and HF-NET on the mortality of patients with CS remains unclear, but symptoms and quality of life might improve [48, 49].

## Limitations

We present data derived from the German governmental coding system. By nature of this study, our data are descriptive. While data for reimbursement-relevant characteristics like MCS usage are highly accurate, non-reimbursement-relevant characteristics like accompanying baseline characteristics might be underreported. The superiority of one MCS device over another or over medical therapy cannot be derived from data presented. Also, we cannot compare patient groups (with and without different MCS devices) to another since a significant selection bias has to be presumed. An adjustment of potential confounders cannot be reasonably performed, since data on disease severity at the time of the treatment decision (like lactate; catecholamine treatment; time, duration and quality of CPR with respect to MCS implantation; experience of the physician and his team; patient wish; necessity of pulmonary support with respect to MCS implantation; heart rhythm; PCI success rate or complications; etc.) are not supplied by the DESTATIS dataset. In the dataset supplied used for this research, the cause of CS is not encoded. We therefore cannot differentiate between different subcategories of CS. Also, we have no information concerning the rationale for using a specific MCS. Thus, we can only speculate why different devices were implanted. Different protocols may have led to a selection bias for or against a given MCS. Maybe, only one method was available in some settings or operators were only trained in one method. As reimbursement differs between the support devices, we cannot exclude a bias by economic considerations. We, further, cannot identify combination therapies or subsequent use of different MCS, which may bias survival data. Results may differ in other high-income countries due to differences in the available intensive care resources or reimbursement situations. The etiology of cardiogenic shock and the supporting medical therapy like inotropic therapy cannot be characterized. The data do not allow us to draw any conclusions regarding long-term survival or quality of life.

## Conclusions

The reported incidence of CS is significantly increasing. Hospital survival rate is low and stagnant. Only a minority of patients with CS is managed with MCS. The type of MCS device used in CS has shifted from the exclusive use of IABP to a variety of pVAD, VA-ECMO and IABP and combination therapies. Now, the use of various devices results in different survival rates. This should inspire further investigation to deliver the most suitable technical option with the best chances of survival for different etiologies of CS.

## Electronic supplementary material

Below is the link to the electronic supplementary material.

Supplementary file1 (DOCX 20 KB)

## Data Availability

The datasets used during and/or analyzed during the current study are available from the corresponding author on reasonable request.

## Abbreviations

ACS: Acute coronary syndrome
AMI: Acute myocardial infarction
CAD: Coronary artery disease
CO: Cardiac output
CPU: Chest pain unit
CS: Cardiogenic shock
DESTATIS: Research Data Center of the Federal Bureau of Statistics in Germany
DRG: Diagnosis-related group
ELSO: Extracorporeal life support organization
HFU: Heart failure unit
HF-NET: Heart failure network
IABP: Intra-aortic balloon pump
ICD: International Statistical Classification of Diseases and Related Health Problems
MCS: Mechanical circulatory support
OPS: German procedure classification
pVAD: Percutaneous ventricular assist device
SAVE-Score: Survival after veno-arterial ECMO
VA-ECMO: Veno-arterial extracorporeal membrane oxygenation
VSD: Ventricular septum defect
