# Supplementary material for: Cardiogenic shock: incidence, survival and mechanical circulatory support usage 2007–2017-insights from a national registry
Source: Clin Res Cardiol. 2020 Nov 30;110(9):1421–30. doi: 10.1007/s00392-020-01781-z (PMC8405485; doi:10.1007/s00392-020-01781-z)
Supplement: Supplementary file 1 — Supplementary file1 (DOCX 20 KB) [file 392_2020_1781_MOESM1_ESM.docx]

Electronic supplemental material

Supplemental Table 1: patient characteristics and comorbidities for pVAD, IABP and VA-ECMO

|  | **Total** | **Medical therapy** | **any MCS** | **PVAD** | **IABP** | **VA-ECMO** |
| --- | --- | --- | --- | --- | --- | --- |
| N | 383,983 | 333,459 | 50,524 | 3,945 | 36,805 | 9,774 |
| Age in years | 71.3 | 72.2 | 65.5 | 65.0 | 67.0 | 60.0 |
| Female | 147,711 (38.5%) | 133,426 (40.0%) | 14,286 (28.3%) | 1,026 (26.0%) | 10,607 (28.8%) | 2,653 (27.1%) |
| NYHA III or IV | 157,431 (41.0%) | 128,741 (38.6%) | 28,690 (56.8%) | 2,577 (65.3%) | 20,533 (55.8%) | 5,580 (57.1%) |
| Hypertension | 141,339 (36.8%) | 121,935 (36.6%) | 19,404 (38.4%) | 1,442 (36.6%) | 14,669 (39.9%) | 3,293 (33.7%) |
| Previous MI | 25,562 (6.7%) | 22,016 (6.6%) | 3,546 (7.0%) | 269 (6.8%) | 2,644 (7.2%) | 633 (6.5%) |
| Previous CABG | 24,102 (6.3%) | 20,506 (6.2%) | 3,596 (7.1%) | 185 (4.7%) | 2,634 (7.2%) | 777 (8.0%) |
| Previous cardiac surgery | 33,448 (8.7%) | 28,661 (8.6%) | 4,787 (9.5%) | 225 (5.7%) | 3,362 (9.1%) | 1,200 (12.3%) |
| PAD | 28,254 (7.4%) | 24,339 (7.3%) | 3,915 (7.8%) | 269 (6.8%) | 2,853 (7.8%) | 793 (8.1%) |
| CAD | 179,740 (46.8%) | 140,581 (42.2%) | 39,159 (77.5%) | 3,082 (78.1%) | 30,043 (81.6%) | 6,034 (61.7%) |
| Carotid artery disease | 6,236 (1.6%) | 4,968 (1.5%) | 1,268 (2.5%) | 50 (1.3%) | 980 (2.7%) | 238 (2.4%) |
| COPD | 38,772 (10.1%) | 35,072 (10.5%) | 3,700 (7.3%) | 245 (6.2%) | 2,776 (7.5%) | 679 (7.0%) |
| Pulmonary hypertension | 28,302 (7.4%) | 23,563 (7.1%) | 4,739 (9.4%) | 349 (8.9%) | 3,081 (8.4%) | 1,309 (13.4%) |
| Renal disease GFR <15% | 14,860 (3.9%) | 13,611 (4.1%) | 1,249 (2.5%) | 111 (2.8%) | 902 (2.5%) | 236 (2.4%) |
| Renal disease GFR <30% | 20,210 (5.3%) | 18,575 (5.6%) | 1,635 (3.2%) | 126 (3.2%) | 1,228 (3.3%) | 281 (2.9%) |
| Atrial fibrillation | 121,074 (31.5%) | 105,179 (31.5%) | 15,895 (31.5%) | 1,186 (30.1%) | 11,371 (30.9%) | 3,338 (34.2%) |
| Diabetes | 116,314 (30.3%) | 100,872 (30.3%) | 15,442 (30.6%) | 1,169 (29.6%) | 11,762 (32.0%) | 2,511 (25.7%) |

Table 1: Patient characteristics and comorbidities grouped in pVAD, IABP and VA-ECMO patients is depicted and compared to the total numbers of medically treated patients and patients on any mechanical circulatory support system (MCS). Case numbers are followed by the percentage.

Supplemental Table 2: Incidence and survival in reported cardiogenic shock 2007-2017, yearly

|  | 2007 |  | 2008 |  | 2009 |  | 2010 |  | 2011 |  | 2012 |  | 2013 |  | 2014 |  | 2015 |  | 2016 |  | 2017 |  |
| --- | --- | --- | --- | --- | --- | --- | --- | --- | --- | --- | --- | --- | --- | --- | --- | --- | --- | --- | --- | --- | --- | --- |
|  | **N** | **survival** | **N** | **survival** | **N** | **survival** | **N** | **survival** | **N** | **survival** | **N** | **survival** | **N** | **survival** | **N** | **survival** | **N** | **survival** | **N** | **survival** | **N** | **survival** |
| whole cohort | 26,828 | 39.2% | 27,492 | 38.9% | 28,666 | 39.4% | 30,067 | 40.3% | 32,294 | 41.0% | 34,670 | 40.3% | 36,473 | 39.4% | 38,649 | 40.0% | 41,269 | 39.9% | 43,150 | 41.1% | 44,425 | 41.2% |
| medical therapy | 23,118 | 38.0% | 23,059 | 37.1% | 23,745 | 37.3% | 24,934 | 37.9% | 27,229 | 39.3% | 29,967 | 39.0% | 32,389 | 39.2% | 34,542 | 40.0% | 37,081 | 40.1% | 38,385 | 41.4% | 39,010 | 41.6% |
| Any MCS | 3,710 | 46.6% | 4,433 | 48.0% | 4,921 | 49.7% | 5,133 | 5.0% | 5,065 | 50.0% | 4,703 | 48.6% | 4,084 | 41.0% | 4,107 | 39.8% | 4,188 | 38.5% | 4,765 | 38.1% | 5,415 | 38.6% |
| pVAD |  |  | 55 | 36.4% | 65 | 32.3% | 76 | 31.6% | 43 | 37.2% | 86 | 32.6% | 219 | 26.9% | 358 | 36.6% | 574 | 35.2% | 980 | 37.1% | 1,489 | 37.6% |
| IABP | 3,676 | 46.8% | 4,315 | 48.5% | 4,759 | 50.4% | 4,943 | 52.8% | 4,861 | 50.8% | 4,350 | 50.2% | 2,758 | 46.8% | 2,287 | 47.7% | 1,826 | 47.4% | 1,607 | 47.8% | 1,423 | 50.9% |
| VA-ECMO | 34 | 23.5% | 63 | 23.8% | 97 | 23.7% | 114 | 28.9% | 161 | 30.4% | 267 | 28.5% | 1,107 | 29.4% | 1,462 | 28.2% | 1,788 | 30.6% | 2,178 | 31.5% | 2,503 | 32.1% |

Table 2: The case numbers of yearly cardiogenic shock incidences from 2007-2017 as well as the total case numbers are shown. Additionally, the survival of the different cohorts is displayed in percentage; grouped in medical or MCS therapy with subgroups of pVAD, IABP and VA-ECMO.
